# Genome-wide epigenetic analyses in Japanese immigrant plantation workers with Parkinson’s disease and exposure to organochlorines reveal possible involvement of glial genes and pathways involved in neurotoxicity

**DOI:** 10.1186/s12868-020-00582-4

**Published:** 2020-07-10

**Authors:** Rodney C. P. Go, Michael J. Corley, G. Webster Ross, Helen Petrovitch, Kamal H. Masaki, Alika K. Maunakea, Qimei He, Maarit I. Tiirikainen

**Affiliations:** 1 Pacific Health Research and Education Institute, 3375 Koapaka Street, Suite I-540, Honolulu, HI 96819 USA; 2 Kuakini Health Systems, 347 N Kuakini St, Honolulu, HI 96817 USA; 3 Department of Epidemiology, School of Public Health, University of Alabama at Birmingham, 1665 University Blvd, Birmingham, AL 35294 USA; 4 Department of Native Hawaiian Health, John A. Burns School of Medicine, University of Hawai’i at Manoa, 650 Ilalo St, Honolulu, HI 96813 USA; 5 Veterans Affairs Pacific Islands Health Care System, 459 Patterson Rd, Honolulu, HI 96819 USA; 6 Department of Geriatric Medicine, John A. Burns School of Medicine, University of Hawaii at Manoa, 650 Ilalo St, Honolulu, HI 96817 USA; 7 University of Hawaii Cancer Center, University of Hawaii at Manoa, 701 Ilalo St, Honolulu, HI 96813 USA

**Keywords:** Parkinson’s disease, Organochlorines, Plantation work, Genome-wide DNA methylation, Glia, Neuroinflammation, Mitochondrial dysfunction, Neurodegeneration

## Abstract

**Background:**

Parkinson’s disease (PD) is a disease of the central nervous system that progressively affects the motor system. Epidemiological studies have provided evidence that exposure to agriculture-related occupations or agrichemicals elevate a person’s risk for PD. Here, we sought to examine the possible epigenetic changes associated with working on a plantation on Oahu, HI and/or exposure to organochlorines (OGC) in PD cases.

**Results:**

We measured genome-wide DNA methylation using the Illumina Infinium HumanMethylation450K BeadChip array in matched peripheral blood and postmortem brain biospecimens in PD cases (n = 20) assessed for years of plantation work and presence of organochlorines in brain tissue. The comparison of 10+ to 0 years of plantation work exposure detected 7 and 123 differentially methylated loci (DML) in brain and blood DNA, respectively (*p *< 0.0001). The comparison of cases with 4+ to 0–2 detectable levels of OGCs, identified 8 and 18 DML in brain and blood DNA, respectively (*p* < 0.0001). Pathway analyses revealed links to key neurotoxic and neuropathologic pathways related to impaired immune and proinflammatory responses as well as impaired clearance of damaged proteins, as found in the predominantly glial cell population in these environmental exposure-related PD cases.

**Conclusions:**

These results suggest that distinct DNA methylation biomarker profiles related to environmental exposures in PD cases with previous exposure can be found in both brain and blood.

## Background

Parkinson’s disease (PD) is a debilitating and prevalent neurodegenerative disease. Increasing evidence suggests a multifactorial biological etiology involving both genetic and environmental predisposing factors that increase risk for PD. Genome-wide association studies have identified a genetic contribution to PD [1, 2] and epidemiological studies have implicated environmental factors such as high toxic exposure to pesticides and herbicides to associate with PD [3]. Initial epigenetic DNA methylation studies in brain tissues used a candidate gene approach to first examine a PD gene alpha-synuclein (*SNCA*) that was found to be associated in high-risk families and known to be abnormally expressed in PD subjects. These studies [4, 5] found that a CpG island demethylation in intron 1 increased the expression of *SNCA.*

More recent studies taking advantage of genome-wide profiling methylation protocols have identified several differentially methylated loci in PD patients versus controls. The epigenome-wide study by Moore et al. [6], selecting a subset of their top hits to follow-up on in a replication set of PD cases versus controls, found two genes with significant differential methylation, Fanconi anaemia complementation group C (*FANCC*) and Tyrosine Kinase Non Receptor 2 (*TNKS2*), which, respectively, are involved in neuronal apoptosis and post-translational signaling. Horvath and Ritz [7], using methylation patterns in 353 CpG sites in the human genome, as an epigenetic age clock [8], were able to show that blood DNA in PD subjects had an increased age acceleration pattern compared to controls. In addition to genomic DNA differential methylation, mitochondrial DNA methylation has also been examined, where a loss of mitochondrial DNA 5-methylation levels was found in the *substantia nigra* (SN) of PD patients by Blanch et al. [9].

Moreover, emerging research has found dysregulated epigenetic mechanisms including altered DNA methylation in postmortem brain specimens as well as peripheral blood leukocytes of PD cases as compared to controls. Masliah et al. [10] examining genome-wide DNA methylation patterns in the frontal cortex and blood from PD patients, found similar patterns of hypomethylation in brain and blood, and reported that *MAPT*, the gene encoding tau, a protein associated with several neurodegenerative diseases, including PD, contained one of their most biologically significant DML. These epigenome-wide PD studies have indicated that using a genome-wide methylation approach may provide insights as to which genes and pathways are not only affected in the development of PD but may also be involved in increasing an individual’s risk to PD given documented occupational and/or environmental exposure to pesticides and herbicides. Also, these recent findings suggest that dysregulated epigenetic mechanisms including DNA methylation may provide biomarkers linking environmental factors and PD. However, few studies have identified key DNA methylation differences in brain and blood associated with specific environmental exposures and neurodegenerative processes involved in PD pathology.

Epidemiological studies have provided evidence for a link between environmental factors and PD [3], and exposure to agrichemicals such as OGC pesticides has been found to associate with PD risk [11]. Furthermore, elevated OGC levels have been found in brains of PD decedents compared to non-PD controls [12, 13]. The number of detectable OGCs associates with increased dopamine levels found in the caudate and putamen brain regions, and also with higher levels of Lewy body pathology [14, 15]. In addition, the Kuakini Honolulu Heart Program study found that 20+ years of plantation work exposure increased the risk of PD by 90% (70% with 11–20 years) [16].

In this report we focus on the effects of plantation work and OGC exposures on the epigenomes in matched blood and brain tissues obtained from PD cases of the Kuakini Honolulu Heart Program cohort. The Kuakini Honolulu Heart Program cohort was generated from a sample of approximately 200,000 Japanese males who were primarily brought to Hawaii to work as contract laborers on the sugar and pineapple plantations between 1885 and 1924 and were likely exposed to OGC pesticides [17, 18]. We used the Illumina HumanMethylation450K BeadChip to detect the DML significantly different between (1) PD cases with 10+ years of plantation work exposure and those with no exposure to plantation work, and (2) PD cases with 4+ OGCs detected and 0–2 OGCs detected. We also examined DML found in both the brain and blood to identify possible concordant DML for possible use as predisposing biomarkers for PD, related to our exposures. Pathway analyses on the DML were also performed to identify neurotoxic pathways related to plantation work and OGC exposure, that interconnect to neuropathological pathways related to PD.

## Results

### Parkinson’s disease participants

Table 1 shows descriptive statistics of some key epidemiological variables describing 20 total cases diagnosed with PD, grouped by either the plantation work exposure or measured brain organochlorine exposure (13 cases overlap between comparison groups, three cases are included only in the OGC analysis and four only in the plantation exposure analysis). There was a significant difference in years of plantation work exposure and the number of OGCs detected between groups. The ages at death and at the time of blood draw of the subjects in plantation work exposure and OGC exposure groups are also included. The OGC test was considered positive if an OGC was detectable and above the level of calibration, based on the results reported from Research Triangle Institute’s high resolution gas chromatographic analyses of the frozen occipital lobe samples provided on the 21 organochlorines tested. [15]. The median number of OGCs detected was 3, thus a 4+ score means that 4 or more of the 21 organochlorines tested were detectable in the frozen occipital lobe brain tissue and thus above the median. The CASI score refers to a 100-point Cognitive Abilities Screening Instrument developed by Teng et al. [19]. A detailed description of how it has been applied to this cohort can be found on our earlier publication [20]. The BRAAK_PD score (by Braak et al. [21]) is based on immunohistochemical staining of the affected brain regions as well as pathology density analyses [15].

**Table 1:** Demographics of PD participants with cortical brain tissues and blood samples

Group	Grouped by exposure				Grouped by OGCs			
	Exposure 10+ years (n = 4)		Exposure 0 years (n = 13)		OGCs 4+ (n = 4)		OGCs 0–2 (n = 12)	
	Mean ± SD	Range	Mean ± SD	Range	Mean ± SD	Range	Mean ± SD	Range
Years of education	8	8–8	11.8 ± 3.3	7–17	12.8 ± 4.3	7–17	9.5 ± 2.1	7–12
Last CASI score	41.6 ± 22.8	18.6–66.9	67.9 ± 24.4	0–90	53.5 ± 37.6	0–86	58.6 ± 24.4	18.5–90
Age at death (years)	88.8 ± 1.3	87–90	85.1 ± 3.9	79–91	84.5 ± 2.5	82–88	87.7 ± 3.4	81–92
Age at blood draw (years)	84.5 ± 1.7	82–86	80.6 ± 3.4	74–86	80.0 ± 3.6	76–83	83.4 ± 4.0	77–91
Plantation exposure (years)	23.2 ± 11.0	15–38	0	0	0.5 ± 1.0	0–2	6.1 ± 11.5	0–38
OGC exposure (n)	2.0 ± 0.8	1–3	2.5 ± 1.2	0–4	4.3 ± 0.5	4–5	1.7 ± 0.7	0–2
Post mortem interval (hrs)	11.8 ± 5.9	5.3–18.7	20.7 ± 21.1	4.2–65.5	7.4 ± 5.0	4.2–14.8	16.8 ± 16.3	4.9–65.6
Braak PD stage	5.8 ± 0.43	5–6	5.7 ± 0.46	5–6	6.0 ± 0.0	6	3.5 ± 0.5	5–6

### Evaluation of the cell type in the temporal lobe DNA specimen

Since we used DNA from bulk postmortem temporal region brain tissue blocks instead of isolated neuronal tissue or cells, we performed a comparative brain cell type-specific DNA methylation analysis utilizing published DNA methylation data for FACS-sorted glia and neurons from human postmortem brain tissue specimens in order to evaluate the cell type composition of the specimen used for this study [22]. We used the published dataset from the cell epigenotype specific model and generated a list of DNA methylation markers containing 20,000 CpG sites that were glia-specific compared to neuron-specific. Next, we evaluated the same 20,000 CpG sites for all 20 of our postmortem brain tissue samples from cases and included seven postmortem control brains for comparison, and analyzed the correlation to both glia and neuron specific CpG sites. We found a highly significant association with our brain tissue specimens’ methylation profiles to the methylation state of glia cells, suggesting our DNA methylation profiles were predominantly derived from glia cells (Table 2). We observed a much weaker relationship between our brain tissue specimens’ methylation profiles and the published methylation state of neurons. Together, these findings confirmed previous reports of the highly glial composition of the temporal region and suggest our data relate mostly to changes in glia cells. This predominantly glial composition of our brain specimens has importance on the DML and pathways they interconnect to, especially since the *substantia nigra* has tenfold higher microglia density than the average in human brain [23].

**Table 2:** Mean and range of R^2^ showing degree of associations between glial and neuronal methylation sites and the 29 (22 cases and 7 control) brain and blood tissue methylation data

Cell type	Glial		Neuronal	
Tissue source	Mean R^2^	Range	Mean R^2^	Range
Brain	0.837	0.684–0.941	0.039	0.000–0.113
Blood	0.451	0.460–0.440	0.008	0.007–0.009

### Identification of DML between high and low plantation work exposure in brain and blood

We used analysis of covariance (ANCOVA) modeling to identify differentially methylated loci (DML) in blood and brain for PD cases with 0 years of plantation work (n = 13) and PD cases with ten or more years of plantation work (n = 4). The analyses for blood were adjusted for age at blood draw. The analyses for brain tissues were adjusted for age at autopsy (age at death), time to autopsy (post mortem interval), and their interaction.

We identified 94 DML in postmortem brain tissue specimens for plantation work exposure (10+ years vs 0 years) as the single major contrast variable with an unadjusted (without multiple test correction) p < 0.001 (Additional file 1: Table S1 and Additional file 2) and 7 DML with p < 0.0001 (Table 3). The top two annotated DML were related to the Prostaglandin D2 Synthase (*PTGDS*) and Peroxisomal biogenesis Factor 19 (*PEX19*) genes and were hyper-methylated in 10+ years plantation work exposure compared to 0 years, with p-values 3.28E−06 and 4.98E−05 respectively.

**Table 3:** Brain DML (p < 0.0001); PD cases with 10+ vs 0 years of plantation work

PROBESET ID	CHR	CpG location	Gene	p-value	Methylation^a^
cg14608180	9	Body	PTGDS	3.28E−06	+
cg08142821	13	Intergenic		4.30E−05	+
cg17880320	1	TSS1500	PEX19	4.98E−05	+
cg06173536	1	TSS1500	GNG4	6.41E−05	+
cg11706717	1	Body	NID1	7.32E−05	+
cg16514167	11	TSS1500	MICALCL	8.24E−05	+
cg04879348	2	3′UTR; Body	GCC2	9.74E−05	−

^a^ Hyper (+) or Hypo (−) methylation

Analysis of blood specimens with plantation work exposure (10+ years vs 0 years) as the single major contrast variable, identified 788 DML with unadjusted p < 0.001 (Additional file 1: Table S1 and Additional file 2) and 123 DML with an unadjusted p < 0.0001. The top two DML were related to the Wingless-Type MMTV Integration Site Family, Member 16 (*WNT16*) and Ectonucleoside triphosphate diphosphohydrolase 8 (*ENTPD8*) genes, with p-values 9.04E−07 and 1.47E−06, respectively (Table 4).

**Table 4:** Top blood annotated DML (p < 0.0001); PD cases with 10+ vs 0 years of plantation work

PROBESET ID	CHR	CpG location	Gene	p-value	Methylation^a^
cg25608490	7	TSS1500; Body	WNT16	9.04E−07	+
cg12179661	9	5′UTR	ENTPD8	1.47E−06	−
cg08435683	20	3′UTR	SLC23A2	1.51E−06	+
cg02286380	17	Body	ARRB2	1.53E−06	+
cg16395997	1	Body	WDR8	3.82E−06	+
cg00145955	16	Body	QPRT	4.02E−06	−
cg04577162	7	Body	RFC2	4.62E−06	+
cg14689338	5	Body	MYO10	6.05E−06	−
cg03997039	16	Body	QPRT	6.11E−06	−
cg26021714	14	Body	RNASE2	7.65E−06	+
cg14070845	17	Body	SPAG5	8.37E−06	+
cg23688843	X	TSS1500	CCNB3	8.51E−06	−
cg02237172	3	Body	ZMYND10	9.38E−06	+
cg12359315	3	Body	SLC6A20	1.13E−05	+
cg16815625	8	Body	TRAPPC9	1.38E−05	+
cg14177914	1	TSS1500	C1orf61	1.53E−05	+
cg01676795	7	Body	POR	1.63E−05	+
cg05978707	2	Body	SNTG2	1.71E−05	−
cg12871835	6	TSS1500	SGK1	1.75E−05	+
cg13847137	4	Body	PPP2R2C	1.84E−05	−
cg03098589	10	Body	VTI1A	1.95E−05	+
cg18011273	4	Body	SORCS2	1.97E−05	−
cg21699894	20	5′UTR; Body	TOX2	2.11E−05	+
cg13478928	1	Body	LHX8	2.13E−05	+
cg07430820	17	Body; 1stExon; 5′UTR	CHD3	2.16E−05	−
cg06145336	2	TSS1500	HOXD3	2.33E−05	+
cg04172851	6	TSS200; TSS200	XPO5; POLH	2.47E−05	+
cg00114346	7	Body	DNAJB6	2.73E−05	+
cg10763483	13	TSS1500	C13orf33	2.79E−05	−
cg11202380	1	3′UTR	AK2	2.91E−05	+
cg08688629	10	TSS1500	KNDC1	3.00E−05	−
cg07017164	17	Body	MAPT	3.23E−05	−
cg06302533	5	TSS200	PCDHB18	3.59E−05	+
cg14101659	6	Body	EXOC2	3.65E−05	+
cg20134658	20	5′UTR	C20orf11	4.07E−05	−

^a^ Hyper (+) or Hypo (−) methylation

**Fig. 1:**
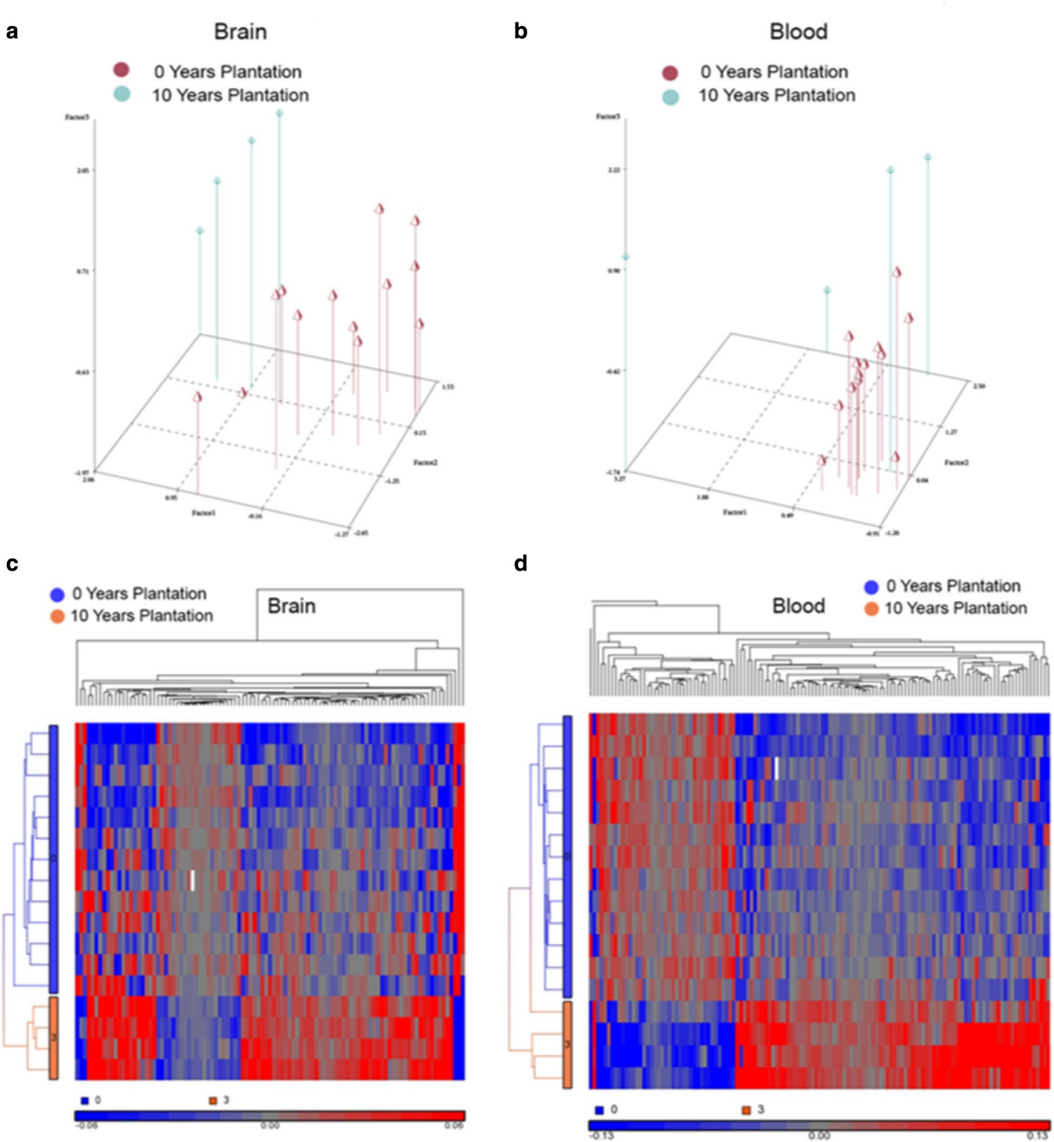
PCA and hierarchical clustering for plantation work DML (10+ years vs 0 years). **a**, **b** Principal component graphs as coordinates in 3-D Euclidian space, depicting the clear separation of the high and low exposure groups. DML loci with p < 0.0001 were used, 7 for brain (**a**) and 123 for blood (**b**). **c**, **d** Hierarchical clustering of the DML for high and low exposure to plantation work in brain and blood. For brain (**c**), 94 DML loci with p < 0.001 were used, for blood (**d**), 123 DML loci with p < 0.0001 were used

We used principal component analyses for the brain and blood derived DML related to plantation work exposure and observed that the high and low exposure groups formed two distinct groups stratified by principal components (Fig. 1a, b). In addition to the principal component analyses (PCA), we used hierarchical clustering of the 94 DML identified in brain specimens and observed that the 4 cases with 10+ years of plantation work exposure (code 3, orange) all clustered together as a group separate from the 13 cases with 0 years of plantation work exposure (Fig. 1c). Next, we used hierarchical clustering of the 123 DML identified in blood specimens and found that all 4 cases with Plantation work exposure of 10+ years (code 3, orange) clustered together on the bottom of the heat map, and were separable from those with 0 years of exposure (code 0, blue) (Fig. 1d). These data on our limited number of samples indicate that work exposures in a person’s younger years, e.g., plantation work exposure here, may associate with altered brain and blood methylation patterns.

### Pathway analyses for plantation work exposure related DML

To examine the exposure-associated DML gene pathways in brain and blood specimens, we created lists of genes with significant DML, using p < 0.001 for brain (94 DML), and p < 0.0001 for blood (123 DML) (different levels of p were used to keep the number of DML from each tissue approximately equal) and performed network and functional classification analysis using the Ingenuity Pathway Analysis (IPA^®^) (Qiagen, Valencia, CA). Additional file 1: Table S2 shows the frequency of the mapping of the DML to unique annotated genes. The 94 DML identified in brain tissue related to plantation work exposure were mapped to 66 unique genes and 58 were analyzable in IPA^®^ (had published inter-connectedness data). In blood, we mapped the DML to 112 unique genes from the 123 DML and 81 were analyzable in IPA^®^. The brain DML pathway analysis revealed ‘neurological disease; cell development, survival and death; and nervous system development and function’ as the top pathways. Moreover, the blood DML detected in comparing those with 10+ years of plantation work exposure to 0 years identified ‘neurological disorders’, e.g., movement disorders, neuromuscular disease, and Parkinson’s disease as top pathways (Additional file 3: Figure S1) The most important genes among the 15 genes with DML in these pathways are Androgen Induced 1 (*AIG1*), glutamate ionotropic receptor NMDA type subunit 2A (*GRIN2A*), B-Cell CLL/Lymphoma 2 (*BCL2*), Serum/Glucocorticoid Regulated Kinase 1 (*SGK1*), and microtubule associated protein gene (*MAPT*).

### Identification of differentially methylated loci (DML) associated with OGC pesticide exposures in brain and blood

ANCOVA modeling was used to identify DML in brain and blood for 12 PD cases with 0–2 detectable vs. 4 cases with 4+ detectable OGCs. The brain tissue analysis identified 69 DML with an unadjusted p-value less than 0.001 (Additional file 1: Table S3 and Additional file 2) and 8 DML with unadjusted p-values < 0.0001 with OGC pesticides exposure (4+ vs 0–2 detectable levels) as the major contrast variable (Table 5). The top two DML in brain were related to the Phosphofurin Acidic Cluster Sorting Protein 2 (*PACS2*) (two loci), at p-values 1.18E−06 and 1.60−05 respectively. The blood sample analysis identified 176 DML with p < 0.001 and 18 DML with p < 0.0001 (Additional file 1: Table S3 and Additional file 2). The top two DML in blood were related to the DnaJ Heat Shock Protein Family (Hsp40) Member C15 (*DNAJC15*) and Adaptor Related Protein Complex 2 Alpha 2 Subunit (*AP2A2*) genes at p-values of 9.55E−06 and 1.10E−05, respectively (Table 6). Notably, two separate DML loci in DNAJC15 were identified in the brain associated with the exposure to the OGCs and they were among the top 8 by p-values.

**Table 5:** Brain DML (p < 0.0001); PD cases with OGC pesticide exposure 4+ vs 0–2 detectable levels

PROBESET ID	CHR	CpG location	Gene name	p-value (Code 2 vs 0)	Methylation^a^
cg18397450	14	Body; Body	PACS2	1.18E−06	+
cg18912855	14	Body	PACS2	1.60E−05	+
cg02101742	13	Body	TFDP1	1.68E−05	−
cg09677945	13	5′UTR; 1stExon	DNAJC15	1.73E−05	+
cg15988970	13	5′UTR; 1stExon	DNAJC15	2.82E−05	+
cg25499461	19	TSS200	IRF2BP1	5.28E−05	+
cg15909600	22	TSS1500	MAPK8IP2	6.08E−05	+
cg06534988	5	Intergenic		8.05E−05	−

^a^ Hyper (+) or Hypo (−) methylation

**Table 6:** Blood DML (p < 0.0001); PD cases with OGC pesticide exposure 4+ vs 0–2 detectable levels

PROBESET ID	CHR	CpG location	Gene name	p-value (Code 2 vs. 0)	Methylation^a^
cg09677945	13	5′UTR; 1stExon	DNAJC15	9.55E−06	+
cg12456927	4	Intergenic		1.10E−05	−
cg02598807	11	Body	AP2A2	1.94E−05	−
cg11667387	10	Body	CNNM2	2.03E−05	−
cg15723784	15	Body; Body; 5′UTR	SMAD3	2.18E−05	+
cg12363545	3	TSS200	CD47	3.24E−05	+
cg16004427	1	Intergenic		3.58E−05	+
cg17242362	7	Body	ATXN7L1	3.71E−05	+
cg12659981	5	TSS1500	ODZ2	4.06E−05	−
cg01047025	9	Body	DMRT1	4.26E−05	+
cg13904560	15	Body	GABRB3	4.53E−05	−
cg10574499	16	TSS200	NRN1L	4.87E−05	+
cg11910782	19	TSS1500	LEUTX	5.10E−05	−
cg10846328	2	Body	BIN1	5.33E−05	−
cg22859727	14	3′UTR	FLRT2	5.97E−05	+
cg26567712	19	TSS200	CCDC124	6.13E−05	+
cg00335735	2	Body	EPHA4	7.07E−05	+
cg10066684	X	TSS1500	TBC1D8B	9.91E−05	+

^a^ Hyper (+) or Hypo (−) methylation

**Fig. 2:**
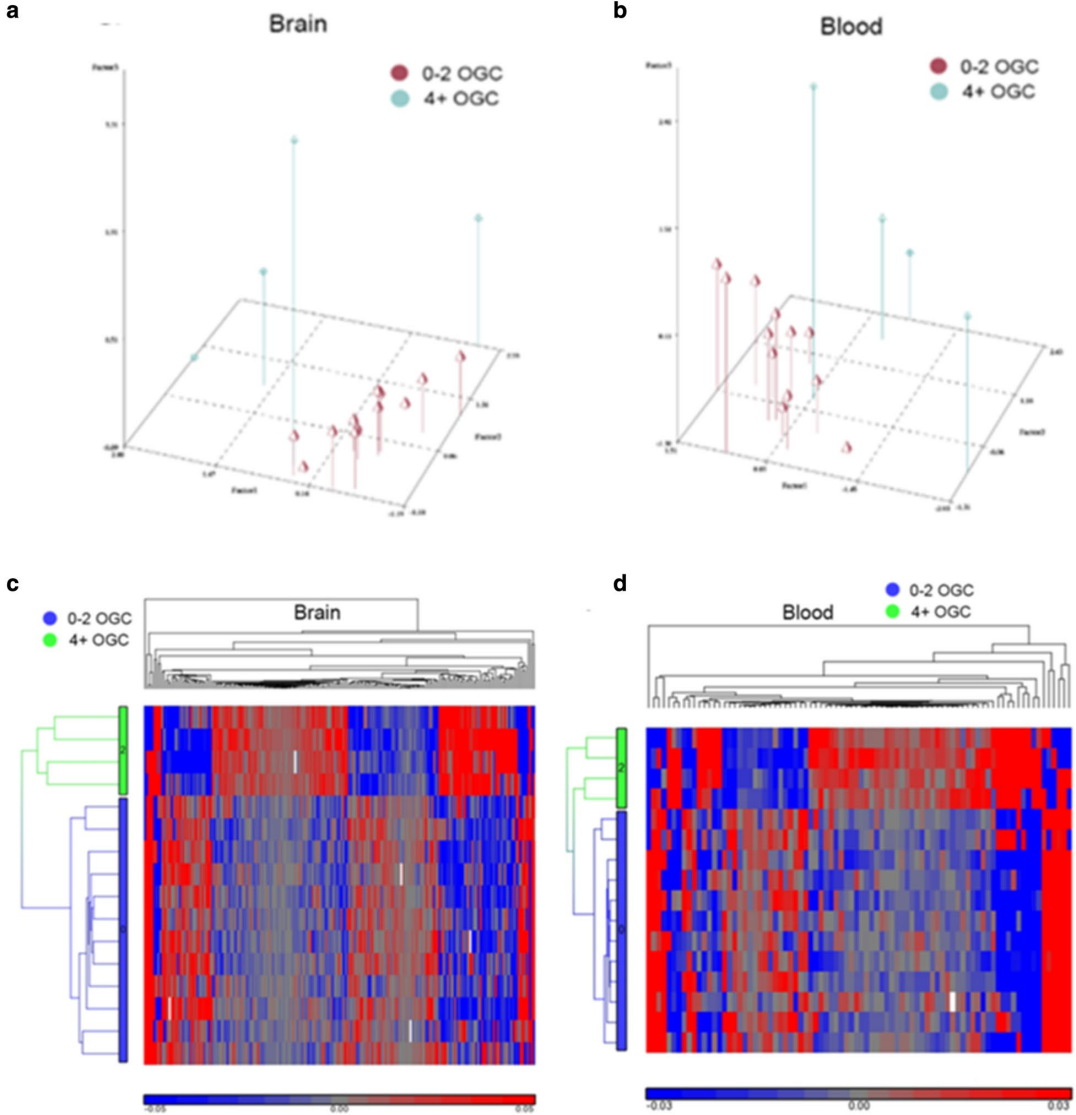
PCA and hierarchical clustering for OGC exposure DML (4+ years vs 0 years). **a**, **b** Principal component graphs as coordinates in 3-D Euclidian space, depicting the clear separation of the high and low exposure groups. DML loci with p < 0.0001 were used, 8 for brain (**a**) and 18 for blood (**b**). **c**, **d** Hierarchical clustering of the DML for high and low detectable OGCs in brain and blood. DML loci with p < 0.001 were used, 69 for brain (**c**) and 176 for blood (**d**)

PCA analysis using the 8 brain DML with p < 0.0001 showed that the 4+ OGC pesticide exposures group was clearly separated from the 0–2 OGC pesticide exposure group (Fig. 2a). Moreover, the PCA analysis of the 18 blood DML with p < 0.0001 also showed distinct separation of the high and low exposure groups (Fig. 2b). In addition, hierarchical clustering analyses of both temporal brain (Fig. 2c) and blood (Fig. 2d) derived DML for 4+ OGC pesticides detected (coded as 2 in green) vs 0–2 detected (coded as 0 in blue) showed that all 4 cases with 4+ detectable pesticides grouped together and formed a separate branch of the cluster tree. These findings suggest that changes in DNA methylation levels at specific genomic loci related to OGC exposures in brain and blood were able to distinguish the PD cases with high (4+) OGC pesticide exposures from those with lower (0–2) OGC pesticide exposures. Whether these altered DNA methylation patterns are shared among those exposed to other pesticides and detectable in other tissues, remains to be examined.

### Pathway analyses for OGC exposure-related DML

Analyses of the DML at p < 0.001 found 69 DML for brain which mapped to 60 unique genes and 176 DML for blood mapped to 136 unique genes, and 53 and 129 were used in IPA^®^, respectively. The top “Diseases and Physiological System Development and Functions” identified by IPA^®^ for DML in blood and brain for the OGC pesticides exposure were related to neurological disease, inflammatory response, and nervous system development. When comparing high and low exposure to OGCs, 27 genes with blood DML were found in pathways related to mitochondrial and neuronal function (Additional file 3: Figure S2), including genes such as Potassium Calcium-Activated Channel Subfamily N Member 3 (*KCNN3*), Microtubule Associated Protein 1B gene (*MAP1B*) and EPH Receptor A4 (*EPHA4*).

### Concordant differentially methylated genes found in brain and blood for plantation work and OGC exposures

Two different DML for *DNAJC15*, both hypermethylated, were differentially methylated in both brain and blood between the OGC exposure groups (Table 7). These loci have been previously identified by Hannon et al. as concordantly methylated between blood and four different brain regions [24]. DNAJC15 protein was described above as having the top blood DML for OGC exposure in our study. Briefly, this gene participates in various cellular processes related to proteins and it also is important to mitochondria.

**Table 7:** Concordant genes associated with OGC exposure in brain and blood

				Brain	Blood
OGC	DNAJC15	cg09677945	+	1.73E−05	9.55E−06
OGC	DNAJC15	cg15988970	+	2.82E−05	9.53E−04

^a^ Hyper (+) or Hypo (−) methylation

We also examined the concordant DML found in both brain and blood samples among those DML that contained common SNPs in the Japanese population at the CpG (filtered out in previous analyses above). The aim was to identify possible SNPs predisposing to neurotoxic processes related to our exposures and PD. The genotype at these CpG loci could possibly be also applicable as a predisposing biomarker for PD (Table 8). We identified one gene with a SNP at the C of the CpG as a concordant DML related to plantation work exposure, *C1QTNF7* (C1q and TNF related 7), which is connected to tumor necrosis factor related apoptosis and is differentially expressed in PD brains. Genotyping of this SNP confirmed its association with methylation status (data not shown).

**Table 8:** Concordant genes with CpG SNPs associated with exposures in brain and blood

Contrast variable	Gene symbol	Probe Id	SNP at CpG	Methylation^a^	p-values	
					Brain	Blood
Plantation work	C1QTNF7	cg18075755	rs13116402	−	5.49E−04	3.28E−04
OGC	DNAJC15	cg19182537	rs2281781	+	7.56E−05	3.02E−04
OGC	CORO7	cg04395006	rs191030943	−	7.80E−04	1.41E−04
OGC	TMCO3	cg05608383	rs117368475	−	4.63E−04	1.72E−04
OGC	MPPED1	cg00740510	rs12483886	−	5.31E−04	9.12E−04
OGC	C14orf182	cg18709904	rs2355655	−	4.36E−04	9.25E−04

^a^ Observed as Hyper (+) or Hypo (−) methylation

Furthermore, we identified 5 concordant genes associated with organochlorines exposure for brain and blood (Table 8), all of them with a common SNP (MAF > 1% in Japanese) at the C of the CpG locus which would cause the actual methylation status of the variant allele to be constitutively unmethylated. Among the 5 concordant DML, there was one more DML for *DNAJC15* (cg19182537, two other ones are in Table 7). Other possibly SNP-derived DML were found in genes for Coronin 7 (*CORO7*), Transmembrane and Coiled-Coil Domains 3 (*TMCO3*), Metallophosphoesterase Domain Containing 1 (*MPPED1*) *andC14Orf182* (Long Intergenic Non-Protein Coding RNA 1588). All of these were also identified by Hannon et al. [24] as blood–brain concordant loci, with the DML locus at *CORO7* showing more concordance between blood and the superior temporal gyrus, compared to the other three brain regions. It is to be noted that the DML CpG locus (and the SNP) for *C1QTNF7, DNAJC15, MPPED1* and *C14Orf182* is in the 5′ region of the gene or just upstream, on a possible promoter region, and we observed a trend for a gene expression pattern to be dependent on the methylation status in brain for *C1QTNF7,DNAJC1 and MPPED1* (data not shown). The DML cg18709904, related to the *C14Orf182 *gene is also aligning with *LINC01588* and *LINC01599*, non-coding RNAs (UCSC Genome Browser GRCh38/hg38 assembly), but the functions of these two LincRNAs are unknown.

### Technical validation of differentially methylated loci

Nine DML identified by the Methylation450K BeadChip were selected for validation using pyrosequencing (Additional file 1: Table S4). Six loci were analyzed using DNA from both the brain tissue and blood. One locus was analyzed in brain only, and two loci were analyzed in blood only. For 6 of the 7 genes tested in Brain DNA, statistically significant correlations (p ≤ 0.002) ranging from 0.57 to 0.90, between the Pyrosequencing and Methylation450K BeadChip, were found, while one DM failed to be validated. For 6 out of 8 loci tested with blood DNA, statistically significant correlations (p ≤ 0.05) with R-values 0.46–0.97 were found.

## Discussion

In this initial study the main goal was to see whether we could identify a set of DML that could distinguish between individuals with Parkinson’s Disease who had high or low levels of two PD associated factors, i.e., plantation work years and detectable OGC pesticide exposures, both previously identified as significant risk factors for PD in the same longitudinal cohort of Japanese-American men [15, 25]. Our results indicated that in both the brain and blood tissue specimens, there were significant differences in DNA methylation related to plantation work and OGC exposures. To examine whether these DML stratified PD cases based on exposure conditions in brain and blood, we used PCA and hierarchical clustering analyses. The results indicated that cases with high and low exposure levels can be distinguished based on brain and blood-specific DNA methylation profiles.

Many of the genes for the DML identified in this study participate in key mechanisms involved in neurodegeneration and PD, i.e., apoptosis, autophagy, mitochondrial dysfunction and mitophagy, oxidative damage and repair, ubiquitin/proteasome system dysfunction, neuroinflammation, axonal development and degeneration, as well as epigenetic processes. The Ingenuity Pathways Analyses revealed that the top affected function is in neurodevelopment, and the IPA^®^ ‘neurological disorders’ pathway contains many of these DML. In particular, we identified DML in PD susceptibility loci, such as those in Parkin RBR E3 ubiquitin protein ligase (*PARK2*) and *MAPT* that were differentially methylated in the comparisons of high and low exposure groups. This is not unexpected given their importance in key pathways related to the hypothesized mechanisms through which exposure to agrichemicals are believed to cause neurodegeneration and death of dopaminergic neurons [26–28], i.e., via neuroinflammation, mitochondrial dysfunction, apoptosis, and ubiquitination/proteasome dysfunction. Below we describe some of these key mechanisms and genes with DML in our study that are related to them.

### Mitochondria dysfunction and neuroinflammation

Many pesticides affect protein complexes in the mitochondrial electron transport chain, and experimental animal models for PD have been developed using compounds, such as 1-methyl-4-phenyl-1,2,3,6-tetrahydropyridine (MPTP), 6-hydroxydopamine (6-OHDA), salsolinol, rotenone, and paraquat. Studies using these animal models have shown that promotion of mitochondrial dysfunction occurs leading to subsequent death of dopaminergic neurons; and all of these neurotoxic models exhibited involvement of activated glial cells [29–31]. For example, it has been shown for MPTP, that a single exposure can induce prolonged and vast microglia activation and increased levels of pro-inflammatory cytokines, tumor necrosis factor-α (TNF-α), and interleukins IL-1α, IL-1β, and IL-6 [32, 33]. These cytokines lead to up-regulation of reactive oxygen species (ROS), nitric oxide (NO), and superoxide radicals, to form highly oxidizing peroxynitrite species and activation of dihydronicotinamide-adenine dinucleotide phosphate (NADPH) oxidase in DA neurons that promotes further oxidative damage and their eventual death through activation of cell death processes in these oxidative stressed neurons (see [34, 90] for review). The brain and in particular the *substantia nigra* is highly sensitive to oxidative stress due to its high oxygen consumption, low antioxidant defenses, high number of oxidizable species, e.g., polyunsaturated fatty acids, iron, and the presence of dopaminergic neurons, which are highly susceptible to oxidative stress, and its pro-oxidative state relative to other brain regions [35]. The brain tissues used in our study was primarily of glial origin, so the results from these animal models implicating activated glia in the neuropathological processes in PD, mirrors our results that focused on OGC and plantation work exposures in PD cases.

Hence mitochondria are central players in apoptotic pathways [36, 37] and are key to the survival of neurons, and are featured in our study in several concordant DML shown in Tables 7 and 8. Key DML identified in our analyses related to mitochondrial dysfunction were in genes encoding for MAPT, MAP1B, SGK1, KCCN3, PACS2, DNAJC15, C1QTNF7, BCL2, CORO7, and TMCO3.

MAPT has been linked to PD and epigenetic studies on PD subjects have reported differences in DNA methylation at the *MAPT* gene [4]. MAP1B, as MAPT, is a microtubule associated protein involved in microtubule assembly. MAPT’s subunit LC1 has microtubule stabilizing activity and it appears to inhibit Leucine Rich Repeat Kinase 2 (LRRK2) activity, interestingly, mutations in *LRRK2* are the most common cause of autosomal dominant and sporadic PD [38]. SGK1 is also linked to MAPT and it codes for a serine/threonine protein kinase that plays an important role in cellular stress response [39]. It phosphorylates MAPT and mediates microtubule depolymerization and neurite formation [40]. Mutations in microtubule associated proteins that lead to hyperphosphorylation and aggregation of tau in brain are linked to a family of neurodegenerative disorders, tauopathies, which leads to destabilization of tau-microtubule interactions leading to instability, axon transport defects, mitochondrial dysfunction, neuroinflammation, and ultimately neuronal death. Mitochondrial dysfunction is a major source of ROS, though more recently microglial cells have also been identified as ROS generators in tauopathies and other neurodegenerative diseases such as PD [41].

Neuroinflammation is closely associated with neuronal degeneration and cell death through biological mechanisms, such as elevated oxidative stress and glial (astrocyte and microglia) cell activation [42, 43]. A DML in this neuroinflammation pathway was found for *MPPED1* which is indirectly connected to SPP1, a PD gene [44] known to be differentially expressed in PD cerebrospinal fluid and brain tissue. SPP1 (OPN), osteopontin, is a glycosylated phosphoprotein expressed in neuronal cell bodies. Osteopontin expression increases after neuronal damage, employing the role of glial cell attractant in this neurodegenerative process [45]. In neurodegenerative disorders, it can be toxic to neurons and cause cell death in some instances, but is neuroprotective in others [46]. A SNP of the osteopontin gene was shown to be associated with Lewy body disease [47]. SPP1’s ties to PD is due to its anti-apoptotic and anti-inflammatory properties and its upregulation in activated microglia which are responding to neuroinflammatory signals [46, 48].

KCNN3, a potassium intermediate small conductance calcium-activated channel (SK) protein, is an integral membrane protein, and it is thought to regulate neuronal excitability by contributing to the slow component of synaptic AHP [49, 50]. A rare intronic SNP for this gene, rs116286121, was found in meta analyses in the PDGENE database [51] to be a significant susceptibility gene for PD with an OR of 1.47 [44]. SK channels have been linked to mitochondrial dysfunction caused by agrichemicals, since it has been shown that activation of SK potassium channels prevents rotenone-induced neuronal cell death and neuronal network degradation, by inhibiting mitochondrial complex I activity [52, 53]. KCCN3 modulates electrophysiological properties of the dopaminergic cells of the *substantia nigra* by regulating the frequency and precision of pacemaker spiking, whereby dysregulation could ultimately lead to altered cell survival signaling pathways [52, 54, 55].

PACS2 is a multifunctional sorting protein that controls the endoplasmic reticulum (ER)-mitochondria communication, including the apposition of mitochondria with the ER and ER homeostasis [56, 57]. Both TMCO3 and PACS2 are involved in communication between the ER and mitochondria [58] through mitochondria-associated membrane (MAM), and hence are important in maintaining normal mitochondrial function. TMCO3 is a Na(+)/H(+) antiporter, i.e., it takes part in the catalysis of the transfer of Na(+)/H(+) from one side of a membrane to the other and is expressed in brain tissues. Most relevant to our exposures’ neurotoxic mechanisms, it Interacts with STX17, which has been shown to be involved in autophagy [59, 60] via a subdomain of the endoplasmic reticulum (ER), called MAM, which forms contacts with mitochondria and determines mitochondria metabolism via the transfer of lipids and Ca^2+^ signals between the ER and mitochondria [58, 61].

BCL2 suppresses apoptosis in a variety of cell systems, including neural cells, by controlling mitochondrial membrane permeability and is a major regulator of neural plasticity and cellular resilience [62, 63]. *BCL2* expression levels were found significantly decreased in brain samples of rotenone treated mice and also in peripheral blood mononuclear cells (PBMC) when comparing PD cases to controls. Both BCL2 and C1QTNF7 play important roles in mitochondrial membrane permeability, apoptosis and cell death. As a member of the complement family of proteins, C1QTNF7 plays a prominent role as a mediator of inflammation in the removal of immune complexes and apoptotic cells, and is a regulator of the immune response, including T-cell responses [64–66]. C1QTNF7 is also part of the interaction network of the BCL-2 athanogene 3 (BAG3), which plays multiple roles in physiological and pathologic processes, including antiapoptotic activity, signal transduction, virus infection, cell adhesion and migration, and initiation of autophagy [67, 68].

CORO7, one of the Coronins, plays a role in Golgi complex morphology and function and interacts with clathrin adaptor AP-1 and is required for the maintenance of Golgi morphology and protein export from the Golgi. It is expressed in high levels in the healthy brain [69, 70]. It has also been shown to be part of the Mitochondrial Protein Import Superpath, which is important for mitochondrial biogenesis and function [71].

### Ubiquitin Proteasome System (UPS) dysfunction

Since many neurodegenerative diseases, such as Parkinson’s disease, involve the accumulation of aberrant and damaged proteins, e.g., a-synuclein and Lewy bodies, and since the Ubiquitin Proteasome System (UPS) is crucial for the degradation of these proteins and hence maintenance of protein homeostasis and normal cell function, reactive gliosis which affects the efficiency of the UPS [72], by promoting proteasome inhibition and neuroinflammation [73], plays a critical role in these diseases. The UPS is a major protein complex involved in the degradation of oxidized proteins, which have been associated with aging and neurodegenerative diseases and is disrupted by organochlorines [74]. It has been shown that mutations in the parkin protein gene *PARK2* are associated with a genetic form of familial PD. Parkin acts as an Ubiquitin ligase in association with proteasomal degradation, and mutations and post-translational modification of this protein causes loss of function of E3 ligase that leads to UPS impairment and the loss of the neuroprotective effects of parkin [75, 76]. Lewy bodies contain not only α-synuclein but also parkin and ubiquitin. Hence in PD, inhibition of the UPS system may contribute to glial dysfunction and subsequent neuronal dysfunction by loss of efficiency in degrading neurotoxic proteins such as α-synuclein (see [73] for review).

The key DML identified in this study linked to the UPS mechanism are in *DNAJC15 * and *PEX19*. DNAJC15 forms a stable subcomplex with a component of the mitochondrial import motor and so participates in the import of proteins into mitochondria, and thus assists in the regulation of the mitochondrial respiratory chain. As a member of the family of J proteins, it also participates, in conjunction with Hsp70 chaperone proteins, in cellular processes, such as folding of proteins, prevention of protein aggregation, disaggregation of proteins and protein transport [40–42]. PEX19 acts as key component of peroxisomes [77] by acting as a chaperone for insertion of peroxisomal membrane proteins (PMPs). The loss of PEX19 results in the absence of detectable peroxisomal structures, destabilization of many integral PMPs, and the mis-localization of other PMPs to the mitochondrion [77].

In eukaryotic cells, mitochondria and peroxisomes are the main ROS contributors [78, 79]. Both are equipped with their own ROS scavenging repertoire of enzymes and hence are key organelles in maintaining cellular ROS homeostasis. Peroxisomes contain several antioxidant systems, which are important for ROS homeostasis, e.g., the β-oxidation pathway, which directly produces H_2_O_2_, which is detoxified by catalase activity and is of central importance for redox balance of the organelle [80, 81]. When peroxisomes are either damaged by excessive ROS production these are marked by ubiquitination, which in mammalian cells is a common signal that triggers autophagy, and pexophagy. Thus, peroxisomal (together with mitochondrial) activity in maintaining the cellular redox state is important for cell survival and health [81–83].

A DML was found also in *AP2A2*; which is involved in pathways resulting in importation of aberrant proteins and toxins into mitochondria and neurons. AP2A2 is a clathrin associated adaptor protein that is involved in clathrin-mediated endocytosis [84] found to be overexpressed in Frontal-Temporal Lobular Dementia [45, 85] and to be important for internalization of alpha synuclein, toxins and neuropathogens into neurons and cells [86, 87].

### Excitotoxicity

Several DML in our study are in genes related to excitotoxicity, neuronal damage and neurodegeneration leading to PD; for example *AIG1*, which is down-regulated in expression studies of PD brains (Nextbio Disease Atlas) [88]. This protein plays an important role in regulating neuron excitability, axonal growth, synaptogenesis and neuronal survival [28, 89]. Another DML is in *GRIN2A*; coding for an *N*-methyl-d-aspartate (NMDA) receptor involved in long-term potentiation, and thought to underlie certain kinds of memory and learning processes. NMDA receptors however play also a critical role in excitatory synaptic transmission and plasticity. Lastly, one of the top DML discovered in blood, related to the plantation work exposure, is in Ectonucleoside triphosphate diphosphohydrolase 8 (*ENTPD8*) gene, which codes for an extracellular enzyme that has phosphohydrolytic activity on ATP and consequent effects on P2-receptor signaling. NTPDase/CD39 ectoenzymes are distributed in the nervous system ubiquitously and they are directly involved in the control of P2 receptor function in nervous tissues [90].

### Aberrant neuronal development

Two DML tied to neurodevelopmental pathways are associated with the Wingless-Type MMTV Integration Site Family, Member 16 (*WNT16*) and *EPHA4*. WNT16 is a member of the WNT gene family that is highly expressed in healthy brain [23, 26]. Wnt ligands modulate expression of target genes that regulate cell proliferation, differentiation, and migration during development of the nervous system [24, 91]. EPHA4 belongs to the ephrin receptor subfamily of the protein-tyrosine kinases. EPH and EPH-related receptors have been implicated in mediating developmental events, particularly in the nervous system [92]. During development the Eph/ephrin system plays a role in the spatial organization of different cell populations, axon guidance, formation of synaptic connections between neurons, and blood vessel remodeling [93–95]. Single nucleotide polymorphisms in various Eph receptors and ephrins have been implicated as modifiers in the pathogenesis of amyotrophic lateral sclerosis as well as Parkinson’s disease [96].

### Concordant DML as biomarkers for exposure-related PD

The studies of Masliah et al. [10] and Davies et al. [97] indicate that use of concordant DML in brain and blood may be important for identification of biomarkers for PD, since these DML may represent the effects of previous environmental exposures that have been flushed from the body, and/or transgenerational effects where environmentally induced changes in epigenetics are passed through the germline [98]. Of the 6 concordant genes with exposure-related DMLs identified in our study (DML with p-values < 0.001 and methylation change in the same direction) between brain and blood for plantation work, only one gene *DNAJC15* had two concordant DML that were not directly SNP-related and one additional DML with a SNP at the CpG, while the other 5 concordant genes all had a DML that contains a common SNP at the CpG that can possibly explain the methylation differences. The value of SNP-related DMLs are however becoming increasingly significant, since the growing number of genome-wide epigenetic (EWAS, like ours here), and mQTL (methylation quantitative trait loci) studies have allowed researchers to combine these data with the GWAS studies to now assign possible function to the many significant SNP signals that have been found to increase risk to diseases. An example is the 5-hydroxytryptamine receptor 2A (5HT2A) genomic variant (T102C) that increases the methylation and decreases expression of the 5HT2A and is hypothesized to be involved in the expression of the schizophrenia phenotype [99]. The proportion of DML found to be related to cis-acting genetic effects vary by phenotype and could be quite high, for example a recent study by Chen et al. [100] found that cis-acting genetic effects could possibly account for as much as 50% of the methylation-expression/phenotype correlation. Hence our 6 concordant DM loci with SNPs could still be significant biomarkers of exposure-related PD, exposure alone, or of populations susceptible to the toxic effects of the neurotoxin exposure, such as experienced by the Japanese male population working on the sugarcane and pineapple plantations in Hawaii.

### Importance of glial origin of methylation signals

Although our brain temporal tissue used for this study was not based on a single cell type, cell composition analyses indicated our methylation signals were primarily glia-derived. Notably, the glial signature was apparent also among the concordant DML in blood as well. The importance of glia to PD pathology has recently become apparent, with the discovery of increased density of activated microglia and astroglia in PD brains and in the brains of genetic, infectious, and environmentally induced animal models of PD. Chronic presence of activated microglia and astroglia is one of the most common features of PD neuropathology and accompanying neurodegeneration [101, 102].

## Study limitations

While our results suggest DNA methylation may provide a biomarker of environmental exposures related to PD, there are limitations to our study. The key limitation of our study is the low number of PD cases analyzed in the high exposure groups, which was due to limited brain autopsy material availability and matched OGC pesticide exposure data. In the design of the study on plantation work and OGC pesticide exposures, a cases-only design was adopted, so as to minimize the identification of genes related to PD susceptibility and pathology only. Another limitation of our study design is the analysis of DNA methylation in bulk brain tissue and blood specimens without focusing on isolated cell types. Previous studies have looked at pesticides dieldrin and paraquat in cultured cells, and noted increased histone acetylation in treated cells [103, 104], however we only investigated a single epigenetic mechanism, DNA methylation, in this study. Moreover, we sampled from only one portion of the brain (temporal), and did not sample from the most affected areas for PD, nor did we compare our results to other relatively unaffected areas of PD brains. Given that our results from the DNA methylation cell type analysis in brain tissue revealed primarily a glial methylation, we assume the results in this study derive largely from this cell type. A further limitation is that the time of exposure for the pesticides in each case cannot be pinpointed to a particular time period in an individual’s life span, and therefore cannot be directly attributed solely to their plantation work history. However, a major strength of this study is that we examined matched blood and brain tissue specimens, taken from the same subjects.

## Conclusions

The finding that our differentially methylated signals were primarily derived from glial rather than neuronal cells is consistent with the known involvement of activated microglia of the M1 type, and astrogliosis in neuroinflammation and neurodegeneration, leading to subsequent PD neuropathology.

The function and network involvement of the discovered DMLs implicated many genes and pathways involved in neurodevelopment, neuroinflammation, neurodegeneration, and the UPS. Most importantly, this epigenomic study revealed that these DMLs activate pathways related to oxidative stress and mitochondrial dysfunction; primary neurotoxic pathways for pesticides and herbicides, e.g., OGCs. Thus, bioinformatics analyses of our significant DMLs, derived primarily from brain tissue of glial origin, supported the importance of glial participation in the neuropathological pathways linked to agrichemical exposure leading to PD.

Our study provides evidence that differences in DNA methylation in both brain and blood, as related to exposure levels, can be found in patients with exposure-related PD. Furthermore, these results support other studies demonstrating association of DNA methylation differences with PD, and also suggest DNA methylation profiles may serve as a biomarker of PD cases with previous work related exposure to pesticides and/or herbicides. Results of this study, however, need to be replicated in a larger cohort and future studies will need to be designed, preferably including systematic genotyping at and around the relevant CpG loci, to determine whether the differences in DNA methylation which are specifically related to these exposures can be attributed to CpG-associated SNPs, at the promoter or enhancer elements and/or at transcription factor binding sites, and so ultimately, are functionally involved in the etiology of PD.

## Methods

### Definition of study population

Originally designed as a longitudinal study of cardiovascular disease, the Honolulu Heart Program (HHP) began in 1965 enrolling 8006 Japanese-American men born 1900–1919 who were living on Oahu, Hawaii [105]. Along with the demographic, lifestyle, dietary, and health related information collected at baseline (1965–1968), study participants were asked if they ever had a regular job on a sugarcane or pineapple plantation and for how many years [25].

Research on PD began in 1991 with the establishment of the Honolulu Asia Aging Study (HAAS) [106]. Previously published standardized methods were used to detect all PD cases in the HAAS beginning at the 1991 examination and continuing in follow-up examinations every 2 to 3 years [107, 108]. The study was approved by the Kuakini Medical Center and the Hawaii Veterans Affairs Institutional Review Boards and participants signed informed consents at all examinations. Since 1991, brain autopsies were sought on all deceased HAAS participants. Consent for autopsy was provided by the closest living relative following Hawaii state law. Hematoxylin and Eosin stained sections of *substantia nigra* (SN) and *locus ceruleus* (LC) were examined by study pathologists, shielded from clinical information, according to a fully standardized gross and microscopic assessment of multiple brain regions as previously reported [16]. In addition, assays for 21 OGC analytes were obtained using dried tissue extracts from frozen occipital lobe samples by high resolution gas chromatography using an electron capture detector [15]. For this analysis, we selected 20 PD cases confirmed by the presence of Lewy bodies in the SN or LC, who were assayed for OGCs, and who had sufficient quantities of frozen brain tissue and blood DNA for genome-wide epigenetic methylation assays.

### Blood collection and DNA extraction

Blood samples were collected by venipuncture at the antecubital fossa during examination 4 (1991–1993), examination 5 (1994–1996) or examination 6 (1997–1999) and stored at − 70 °C. Frozen buffy coat samples were used for extraction of DNA. The PureGene system (Gentra Systems, Inc.) was used to isolate total cellular DNA, which was quantified using PicoGreen staining (Molecular Probes).

Procedures performed were in compliance with institutional guidelines and were approved by the Kuakini Medical Center Institutional Review Board. Written informed consent was obtained at all examinations from study participants or from family representatives if participants were unable to provide consent.

### Nucleic acid isolation from brain tissues

DNA was extracted from fresh frozen brain tissue using the AllPrep DNA/RNA kit (Qiagen, Inc). The extracted nucleic acids were stored at − 80 °C until used.

### Genome-scale methylation profiling

Methylation profiling was performed using the Illumina Infinium methylation assay and the Human Methylation450K BeadChip that interrogates approximately 480,000 CpGs and covers 99% of RefSeq NM and NR genes and 96% of CpG islands.

Five-hundred ng of DNA were treated with bisulfite using EZ-96 DNA methylation kit (Zymo Research), and 100–200 ng of bisulfite-converted DNA was hybridized onto the Infinium Human Methylation450K BeadChip, following the manufacturer’s Infinium HD Methylation protocol. The BeadChips were scanned on the Illumina iScan and the intensities of the images were extracted using the Illumina GenomeStudio Methylation module software.

The methylation score for each CpG is represented as a Beta value according to the fluorescence intensity ratio. GenomeStudio Methylation module software (Illumina) was used to normalize the data to multiple internal controls with background subtraction. Every Beta value on the Methylation450K BeadChip is accompanied by a detection p-value indicating signals significantly greater than background, and probes with p-value higher than 0.05 were filtered out.

### Technical validation of differential methylation

A subset of DML (n = 9) found by the Methylation450K BeadChip analysis were selected for technical validation using Pyrosequencing on a PyroMark Q24 instrument (Qiagen). Bisulfite treated DNA was first amplified by PCR in the regions of interest using the PyroMark PCR kit following the suggested protocol. All pyrosequencing assays matched the DML on the BeadChip and were ordered from Qiagen (three pre-designed and 6 custom designed; designs are available upon request). The pyrosequencing was performed using the PyroMark Gold Q24 Reagents. Purification and subsequent processing of the biotinylated single-stranded DNA were performed according to the manufacturer’s recommendations. The sequencing results were analyzed using the PyroMark Q24 software.

### Statistical analysis of differential methylation

Partek Genomics Suite (Partek, Inc.) was used for the statistical analysis of differential methylation. The probes were filtered for those with SNPs within the 5 bases of the methylation interrogation site; for probes at repeats or that had mapping or copy number issues; (or other technical issues), that were specific for Japanese population when applicable, using a masking strategy suggested by Zhou et al. [109].

Data quality control analyses on the normalized average Beta values generated by the GenomeStudio included Principal component analysis (PCA) of the 20 blood and brain tissue samples, and graphing the sample histograms for signal distributions (data not shown) to distinguish blood from brain tissue samples. For the actual differential methylation analysis, a multivariate analysis of co-variance (ANCOVA), including factors such as tissue type (brain vs. blood), OGC levels, plantation work exposure, age at blood draw (for blood), age at death (autopsy) and time to autopsy (post mortem interval, for brain tissue), was performed to evaluate the contribution of these factors to the differences in methylation (as sources of variation). The full models of the analyses performed are presented on Additional file 2. Heat maps, based on hierarchical clustering methods, were also generated using the Partek Genomics Suite.

In addition to the initial PCA analyses for the QC, the beta values in those loci with p-values < 0.0001 were analyzed in PCA analysis. Three principal components were calculated. Using the three principal component values as the coordinates in three-dimensional (3-D) Euclidian space, the positions of the high (blue) and low exposure groups (red) were plotted, herein referred to as the PCA exposure graphs.

### Ingenuity Pathway Analysis

The Ingenuity Pathway Analysis (IPA^®^) software was used to identify the possibly affected gene networks, functional categories and canonical pathways related to Parkinson’s disease, including the associated genes, and neural functions. IPA^®^ ranks gene networks by a score (− log (p-value)) that takes into account the number of focus genes and the size of the network.

### Cell composition analysis

The Cell EpigenoType Specific (CETS) mapper R package was used to acquire the mean beta values of 20,000 most differentially methylated loci distinguishing FACS-sorted neurons and glia in postmortem human brain control tissue samples [22]. These brain cell type specific loci were identified in the Human Methylation450K BeadChip datasets acquired for our PD postmortem brain samples and correlated to the mean beta values that distinguished neurons and glia.

## Supplementary information

**Additional file 1: Table S1.** Distribution of DML in brain and blood between PD cases with 0 years of plantation work (n = 13) and PD cases with 10+ years of plantation work (n = 4). **Table S2.** DML with p < 0.001 and the number of associated annotated genes. **Table S3.** Distribution of DML in brain and blood between PD cases with 4+ OGCs (n = 4) and PD cases with 0–2 OGCs (n = 12) detected in brain tissue. **Table S4.** HumanMethylation450K BeadChip Analysis and Pyrosequencing correlation.

**Additional file 2.** ANCOVA models used and Annotated lists of all DML with p < 0.001 from ANCOVA analysis. DML with p < 0.0001 are in bold. SNPs and their distances from the query base are indicated as well as the masking suggestions by Zhou et al for the Japanese population (based on SNPs w/in last 5 bases and other possible probe issues including mapping accuracy. FALSE = no issues).

**Additional file 3: Figure S1.** IPA® gene and function interconnection network of 15 genes with blood DML (p < 0.0001) that are associated with PD and other similar disorders, as derived from the comparison of PD cases with Plantation Work 10+ vs 0 years. **Figure S2.** IPA® gene and function interconnection network of 27 genes with blood DML (p < 0.001) that are associated with neurological development and abnormal brain morphology, as derived from the comparison of PD cases with 4+ OGCs and vs 0–2 OGCs.

## Data Availability

The datasets supporting the conclusions of this article are included within the article and its additional files. The methylation array raw data and applicable clinical parameters for this study can be accessed at the NCBI Gene Expression Omnibus (GEO) under Accession number GSE151355. The custom pyrosequencing assay designs are available upon request.

## Abbreviations

PD: Parkinson’s disease
OGC: Organochlorine
SN: Substantia nigra
DML: Differentially methylated loci
ANCOVA: Analysis of covariance
PCA: Principal component analysis
UPS: Ubiquitin Proteasome System
MAPT: Microtubule-Associated Protein Tau
PACS2: Phosphofurin Acidic Cluster Sorting Protein 2
DNAJC15: DnaJ Heat Shock Protein Family (Hsp40) Member C15
