# Supplementary material for: Genome-wide epigenetic analyses in Japanese immigrant plantation workers with Parkinson’s disease and exposure to organochlorines reveal possible involvement of glial genes and pathways involved in neurotoxicity
Source: BMC Neurosci. 2020 Jul 10;21:31. doi: 10.1186/s12868-020-00582-4 (PMC7350633; doi:10.1186/s12868-020-00582-4)
Supplement: Supplementary file 1 — Additional file 1: Table S1. Distribution of DML in brain and blood between PD cases with 0 years of plantation work (n = 13) and PD cases with 10+ years of plantation work (n = 4). Table S2. DML with p < 0.001 and the number of associated annotated genes. Table S3. Distribution of DML in brain and blood between PD cases with 4+ OGCs (n = 4) and PD cases with 0–2 OGCs (n = 12) detected in brain tissue. Table S4. HumanMethylation450K BeadChip Analysis and Pyrosequencing correlation. [file 12868_2020_582_MOESM1_ESM.docx]

**Supplemental Table 1. Distribution of DML in brain and blood between PD cases with 0 years of Plantation Work (n=13) and PD cases with 10+ years of Plantation Work (n=4)**

| **Description of Feature** | **Brain** | **Blood** |
| --- | --- | --- |
| Differentially Methylated Loci Beta Values p<0.001 (p<0.0001) | 94 (7) | 788 (123) |
| Increased Methylation | 69 (6) | 495 (87) |
| Autosomal | 70 (6) | 485 (85) |
| Annotated Genes | 46 (5) | 604 (93) |
| Decreased Methylation | 25 (1) | 296 (36) |
| Autosomal | 25 (1) | 283 (34) |
| Annotated Genes | 20 (1) | 187 (19) |

**Supplemental Table 2. DML with p<0.001 and the number of associated annotated genes**

| Comparison | DML | Annotated Genes | |  | Genes Analyzable in IPA^®^ | |  |  |  |
| --- | --- | --- | --- | --- | --- | --- | --- | --- | --- |
| Brain; Plantation Work 10+ years vs 0 | 94 | 66 | |  | **58** | |  |  |  |
| Blood; Plantation Work 10+ years vs 0 | 123* | 112 | |  | **81** | |  |  |  |
| Brain; 4+ OGCs vs 0-2 OGGs | 69 | 60 | |  | **53** | |  |  |  |
| Blood; 4+ OGCs vs 0-2 OGGs | 176 | 136 | |  | **129** | |  |  |  |
| *p<0.0001 |  |  |  | | |  | |  |  |

**Supplemental Table 3. Distribution of DML in brain and blood between PD cases with 4+ OGCs (n=4) and PD cases with 0-2 OGCs (n=12)** **detected in brain tissue**

| Description of Feature | Brain | Blood |
| --- | --- | --- |
| Differentially Methylated Loci p<0.001 (p<0.0001) | 69 (8) | 176 (18) |
| Increased Methylation | 49 (6) | 106 (11) |
| Autosomal | 48 (6) | 101 (10) |
| Annotated Genes | 46 (4) | 84 (10) |
| Decreased Methylation | 20 (2) | 70 (7) |
| Autosomal | 18 (2) | 67 (7) |
| Annotated Genes | 14 (1) | 52 (6) |

**Supplemental Table 4. Meth450K BeadChip Analysis and Pyrosequencing correlation**

| CpG locus | Gene | Tissue | Correlation^*^ | p-value^$^ | n |
| --- | --- | --- | --- | --- | --- |
| cg00740510 | MPPED1 | Brain | 0.9 | <0.0001 | 26 |
| cg00740510 | MPPED1 | Blood | 0.86 | <0.0001 | 18 |
| cg09677945 | DNAJC15 | Brain | 0.57 | 0.002 | 27 |
| cg09677945 | DNAJC15 | Blood | -0.06 | 0.81 | 18 |
| cg12923728 | SDHAP1 | Brain | 0.58 | 0.002 | 28 |
| cg12923728 | SDHAP1 | Blood | 0.36 | 0.38 | 8 |
| cg24818524 | FRMPD4 | Brain | 0.9 | <0.0001 | 28 |
| cg24818524 | FRMPD4 | Blood | 0.97 | <0.0001 | 19 |
| cg05608383 | TMCO3 | Brain | 0.78 | <0.0001 | 19 |
| cg05608383 | TMCO3 | Blood | 0.46 | 0.05 | 19 |
| cg06065608 | PARK2 | Brain | 0.85 | <0.0001 | 28 |
| cg06065608 | PARK2 | Blood | 0.8 | 0.0004 | 15 |
| cg00044245 | EPHA7 | Brain | -0.08 | 0.67 | 28 |
| cg25608490 | WNT16 | Blood | 0.95 | <0.0001 | 19 |
| cg21699894 | KLRD1-TOX2 | Blood | 0.94 | <0.0001 | 19 |
| ^*^Pearson correlation cofactor R;  ^$^ Two-tailed p-value; n number of samples tested | | | | | |
