# Supplementary material for: Genome-wide epigenetic analyses in Japanese immigrant plantation workers with Parkinson’s disease and exposure to organochlorines reveal possible involvement of glial genes and pathways involved in neurotoxicity
Source: BMC Neurosci. 2020 Jul 10;21:31. doi: 10.1186/s12868-020-00582-4 (PMC7350633; doi:10.1186/s12868-020-00582-4)
Supplement: Supplementary file 3 — Additional file 3: Figure S1. IPA® gene and function interconnection network of 15 genes with blood DML (p < 0.0001) that are associated with PD and other similar disorders, as derived from the comparison of PD cases with Plantation Work 10+ vs 0 years. Figure S2. IPA® gene and function interconnection network of 27 genes with blood DML (p < 0.001) that are associated with neurological development and abnormal brain morphology, as derived from the comparison of PD cases with 4+ OGCs and vs 0–2 OGCs. [file 12868_2020_582_MOESM3_ESM.pptx]

## Slide 1
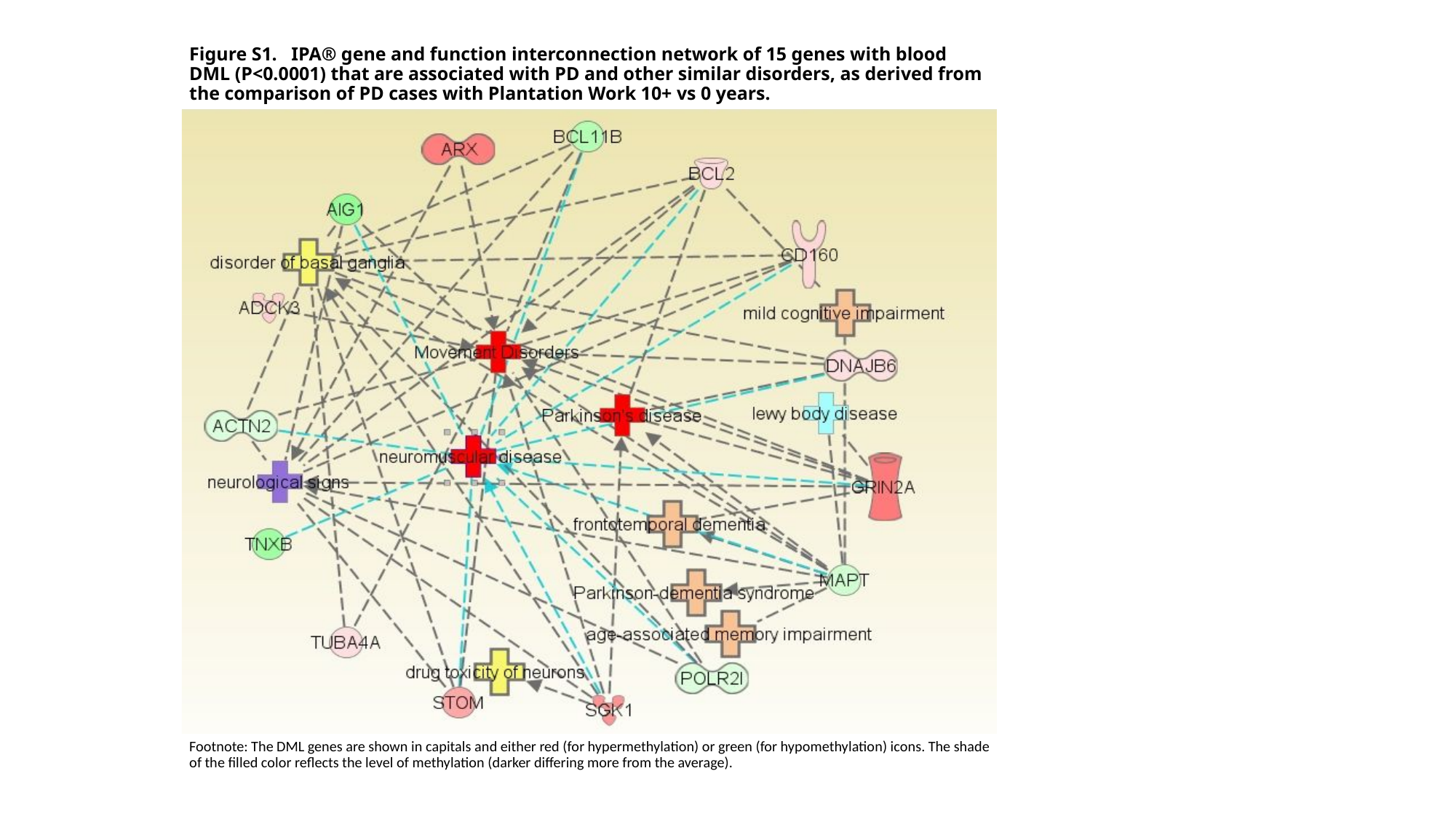

# Figure S1. IPA® gene and function interconnection network of 15 genes with blood DML (P<0.0001) that are associated with PD and other similar disorders, as derived from the comparison of PD cases with Plantation Work 10+ vs 0 years.
Footnote: The DML genes are shown in capitals and either red (for hypermethylation) or green (for hypomethylation) icons. The shade of the filled color reflects the level of methylation (darker differing more from the average).

## Slide 2
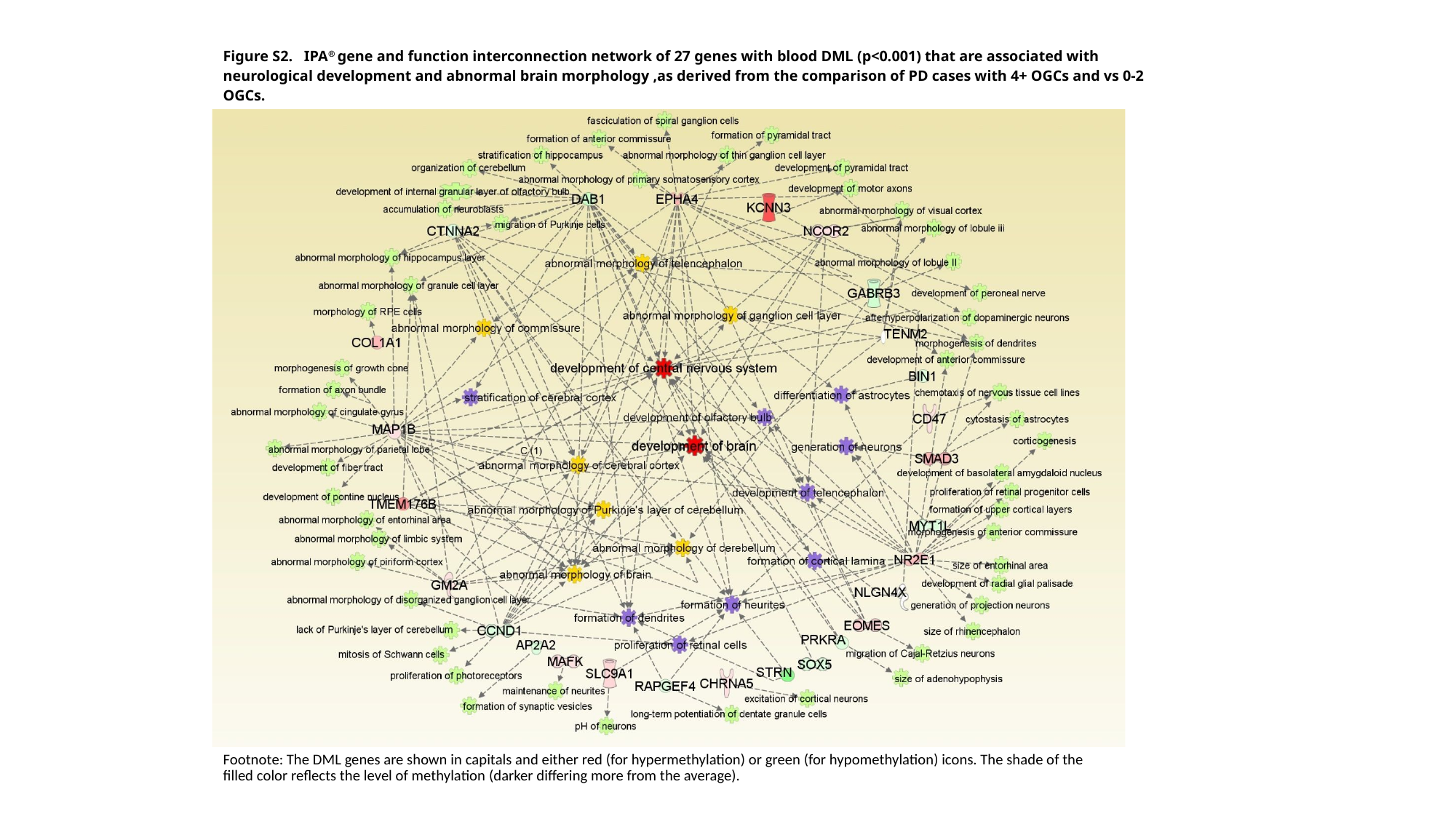

# Figure S2. IPA® gene and function interconnection network of 27 genes with blood DML (p<0.001) that are associated with neurological development and abnormal brain morphology ,as derived from the comparison of PD cases with 4+ OGCs and vs 0-2 OGCs.
Footnote: The DML genes are shown in capitals and either red (for hypermethylation) or green (for hypomethylation) icons. The shade of the filled color reflects the level of methylation (darker differing more from the average).
